# Understanding Caregiver Challenges: A Comprehensive Exploration of Available Resources to Alleviate Caregiving Burdens

**DOI:** 10.7759/cureus.43052

**Published:** 2023-08-06

**Authors:** Bunnarin Theng, Jessica T Tran, Hani Serag, Mukaila Raji, Huey-Ming Tzeng, Miaolung Shih, Wei-Chen (Miso) Lee

**Affiliations:** 1 Radiology, John Sealy School of Medicine, University of Texas Medical Branch, Galveston, USA; 2 Medicine, John Sealy School of Medicine, University of Texas Medical Branch, Galveston, USA; 3 Department of Internal Medicine - Endocrinology, University of Texas Medical Branch, Galveston, USA; 4 Department of Internal Medicine - Geriatrics & Palliative Medicine, University of Texas Medical Branch, Galveston, USA; 5 Department of Preventive Medicine and Population Health, University of Texas Medical Branch, Galveston, USA; 6 School of Nursing, University of Texas Medical Branch, Galveston, USA; 7 Artificial Intelligence, Humanistic Buddhism Practice (HBP), Osher Lifelong Learning Center, University of Texas Medical Branch, Galveston, USA; 8 Department of Family Medicine, University of Texas Medical Branch, Galveston, USA

**Keywords:** gerontology, content analysis, aging, informal care, resources, burden, caregiving

## Abstract

Introduction: Aging is associated with significant alterations in physical, cognitive, and emotional functions, predisposing older adults to multimorbidity and functional dependence that necessitate assistance with the activity of daily living (ADL) and medical care from caregivers. With a substantial increase in the aging population comes a growing demand for caregivers, particularly informal caregivers who provide unpaid care to older adults with complex needs. However, they face substantial physical, emotional, and financial burdens as they balance caregiving with their family and job demands.

Aim: This study aimed to explore key challenges faced by caregivers and the resources they need to address their caregiving burden. Additionally, we wanted to identify whether the number of years of caregiving is associated with their burden. These study findings will inform the design and development of our smartphone app which aims to alleviate the burden of diseases for older adults and the burden of caregiving for caregivers.

Methods: From October to December 2022, we invited 80 self-reported caregivers for an anonymous online survey. The caregivers were located in three cities (Galveston, Houston, and Dallas in Texas) and were affiliated with the International Buddhist Progress Society-Dallas (IBPS Dallas) and the University of Texas Medical Branch (UTMB) Osher Lifelong Learning Institute (OLLI). The collected data were subjected to content analysis through systematic examination for meaningful patterns, themes, and insights. Individual characteristics and caregiving experiences were divided by years of care: 0-4 vs. 5+ years to investigate whether the caregiving burden was affected by the duration of caregiving.

Results: The results showed several important insights, including gender dynamics and traditional norms, the advanced age of caregivers, and the prevalent health conditions they are managing. Caregivers’ roles ranged from medical (insurance and medication assistance, etc.) at 63.8% to the provision of non-medical related resources (basic needs, utility, transportation, financial assistance, etc.) at 96.3%. Caregiving is also associated with some positive outcomes, such as changes in knowledge and skills (77.5%) with more confidence in ADL support tasks and a deepening of caregiver/care recipient dyad relationships (56.3%). Some faced challenges in navigating complex healthcare and social service systems and others experienced neglect and received inadequate support from the government-supported social services (33.8%). However, there is no significant variation between those with 0-4 and 5+ years of experience across all identified themes, suggesting that the burdens and resource needs of caregivers can manifest early on and are likely to persist beyond the five-year mark.

Conclusion: Our findings reveal that the number of caregiving years does not significantly influence the types of caregiving burden experienced by caregivers or the resources they require. This indicates the need for providing long-term support to older adults with chronic conditions from the early stage, while also emphasizing the critical role of immediate assistance for caregivers to alleviate caregiving burden. A free-of-charge technology like our smartphone app has the potential to effectively reduce stress for caregivers, offering them support at any time and place. Future studies will focus on evaluating the outcomes of caregivers after utilizing our app.

## Introduction

Aging is associated with significant alterations in physical, cognitive, and emotional functions, predisposing older adults to increased rates of multimorbidity and functional dependence that may necessitate specialized assistance with activities of daily living (ADLs) and care from caregivers. The care recipients often require long-term support and consistent care to manage their declining health conditions and daily living challenges. In recent years, the world has witnessed a dramatic demographic shift with a substantial increase in older populations. According to the World Health Organization, the estimated global population of individuals aged 60 years and older was one billion in 2019 and is projected to rise to 1.4 billion by 2030 and a staggering 2.1 billion by 2050 [1]. This transformation can be attributed to various factors, such as improved living conditions and advancements in modern medicine, resulting in declining mortality rates and rising life expectancy rates [2]. Consequently, this demographic change poses significant challenges and implications for healthcare systems, social services, and families worldwide [1]. With such a substantial increase in the aging population comes a growing demand for formal and in-kind caregivers [3]. In-kind caregivers provide unpaid care to their loved ones and play a vital role in maintaining the health, well-being, and quality of life of clinically complex older patients living at home [4]. However, the caregivers face substantial physical, emotional, and financial burdens as they balance the myriad demands of caregiving with their own family and job responsibilities [5].

The defining characteristics of an informal caregiver typically include being a person who provides some type of unpaid, ongoing assistance with ADLs or instrumental activities of daily living (IADLs) to a person with a chronic illness or other forms of physical and mental disability [4]. Caregiving responsibilities may also involve emotional support and help with managing a chronic disease or disability [6]. Caregiving responsibilities can increase and change as the recipient’s needs increase, which may result in additional strain on the caregiver [7-9]. Data showed that caregivers are at elevated risk for anxiety, depression, sleep disorders, and psychological distress [10-13].

However, due to rising costs in healthcare and economic challenges, informal caregiving has become an increasingly prevalent and indispensable part of aging populations [14]. One study found that the cost of informal caregiving was estimated to account for 21% of household income [15]. Informal caregivers face numerous challenges as they juggle caregiving responsibilities alongside their health conditions, family commitments, and work obligations, which can lead to significant stress and burden, often resulting in profound exhaustion and severe burnout. These challenges often involve different aspects, such as physical, psychological, and financial burdens, further exacerbating the strain experienced by caregivers.

The overall impact of physical, psychological, social, and financial demands of caregiving has been termed caregiver burden [16]. One study has found that being a caregiver experiencing mental or emotional strain is a risk factor for mortality among elderly spousal caregivers, as those who report strain associated with caregiving are more likely to die than non-caregivers [17]. It has been suggested that the combination of loss, prolonged distress, the physical demands of caregiving, and biological vulnerabilities of older caregivers may compromise their physiological functioning and increase their risk for physical health problems, leading to increased mortality [17]. Furthermore, another study found that chronic stress, such as that experienced by caregivers, can significantly impact the body’s immune system, leading to increased vulnerability to illnesses and diseases that can lead to premature death [18]. They also noted that chronic stress can contribute to other health problems, such as hypertension and cardiovascular disease, which can further increase the risk of mortality [18].

It is imperative to recognize the struggles and challenges faced by caregivers, the impact of caregiving, and the need to provide support and resources to help them manage stress and improve their coping strategies to improve their overall health outcomes and reduce the risk of their care recipients being transitioned to nursing homes. This study was designed to understand and explore the diverse challenges caregivers encounter, gain insights into the burden they perceive, and identify essential resources necessary to sustain and enhance their well-being. Additionally, we aimed to identify whether the number of years of caregiving is associated with their burden. Ultimately, these study findings will inform the design and development of our smartphone app, which aims to alleviate the burden of diseases for older adults and the burden of caregiving for caregivers.

## Materials and methods

Data source

A team from the International Buddhist Progress Society-Dallas (IBPS Dallas) and the University of Texas Medical Branch (UTMB) developed a smartphone application (called utmbHealthyBrain) based on the principle of Humanistic Buddhism to lift the burden of caregivers and improve the well-being of older adults. Humanistic Buddhism emphasizes compassion, mindfulness, and the promotion of overall well-being. It aims to support caregivers with resources and emotional assistance and promote both caregivers' and older adults' mental and physical health through tailored exercises and content that fosters compassion and understanding. Humanistic Buddhism was first introduced by the funder Master Hsing Yun (1927-2023) of Fo Guang Shan, and he published two books that were used for the content of the app: Humble Table (ISBN: 978-1-932293-56-2) and Pearls of Wisdom (ISBN: 0-9717495-6-6). To adapt this app to better meet the needs of caregivers, the project team conducted this caregiver survey among volunteers, and the project was determined as non-human-subject research by the Institutional Review Board at UTMB (#21-0338).

Study sample

The study comprised a convenient sample of caregivers who self-reported caring for one or more individuals aged 50 and older. The project team invited volunteers affiliated with IBPS Dallas or UTMB Osher Lifelong Learning Institute (OLLI) in three different cities: Galveston, Houston, and Dallas, TX. The survey was completely anonymous, and volunteers submitted their responses online. From October to December 2022, there were 84 responses. After removing two incomplete responses, the total valid sample for the analysis is 82 (97.6%).

Measurements

**Figure 1:**
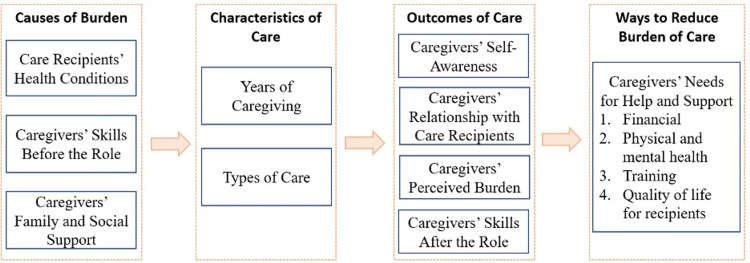
The Overall Framework of This Caregiver Survey

Figure 1 demonstrates the overall framework of this caregiver survey, including four sections (1) causes of burden, (2) characteristics of care, (3) outcomes of care, and (4) suggested strategies to reduce the burden of care. Under the first section, the survey asked respondents to report the health conditions of individuals they care for, their skills and professions, and the support they received. Under the second section, respondents self-reported their years of caregiving and types of care. Following that, respondents described their experiences in four different aspects, such as how their relationship with recipients and what financial burden they have. Finally, respondents were asked about what resources they needed to support and sustain their caregiving.

Analysis

Using the years of caregiving, we divided the sample into two groups: 0-4 years and 5+ years of experience as our independent variable. In the descriptive analysis, numbers, means, percentages, and standard deviations were used to present the characteristics of caregivers, their relationships with recipients, and types of care. Chi-square tests were conducted to compare categorical variables (e.g., gender, race, marital status) between caregivers with 0-4 years and those with more than five years of experience, and two-sample t-tests for numeric variables (e.g., age and number of languages they speak).

In content analysis, 82 responses were categorized by two coders (authors BT and JT) and validated by the author WL to enhance the trustworthiness and credibility of the data analysis. The differences were resolved by discussions to ensure all categories were mutually exclusive. The responses include care recipients’ health conditions (e.g., using wheelchairs), caregivers’ background (e.g., has a job as a nurse), different challenges to care for older adults (e.g., lack of insurance), recommended resources to alleviate the caregiving burden (e.g., a family member’s support). In addition to identifying evolving themes, all responses were compared between caregivers with 0-4 years and those with five or more years of experience. The comparison was reviewed to better understand the association between the length of caregiving and the outcomes of caregiving, but it was not necessary to construct a causal model.

## Results

Of 82 respondents, 43 (52.4%) have 0-4 years of experience and 39 (47.6%) have been caregivers for five years or more. Table 1 shows that their demographic distribution is even (p>0.05), meaning their burden of care or outcome of care would not be influenced by their characteristics. Despite the years of caregiving, the majority (78.1%) of respondents are still in the workforce (70.7% full-time, 3.7% part-time, and 3.7% self-own business). Also, nine in ten caregivers are female, which probably could be explained by the traditional norm that women play the role of caregiver in the family. Finally, around 97.6% of respondents have a smartphone, highlighting the high prevalence of smartphone ownership among caregivers which means the vast majority of them can potentially benefit from this smartphone application. However, the remaining 2.4% of respondents may face challenges in accessing smartphone-based resources and support.​

**Table 1:** Caregiver’s Characteristics by Years of Care

Variables	Total (N=82)	0-4 years (n=43)	5+ years (n=39)	p-value
Age (min, max)	(23, 80)	(23, 80)	(39, 80)	
Age (mean, std.)	56.0 (1.27)	53.9 (1.94)	58.3 (1.52)	0.084
Gender	-	-	-	0.884
Male	8 (9.8%)	4 (9.3%)	4 (10.3%)	
Female	74 (90.2%)	39 (90.7%)	35 (89.7%)	
Race	-	-	-	0.145
White	38 (46.3%)	18 (41.9%)	20 (51.3%)	
Black	9 (11.0%)	7 (16.3%)	2 (5.1%)	
Hispanic	16 (19.5%)	7 (16.3%)	9 (23.1%)	
Asian	16 (19.5%)	11 (25.6%)	5 (12.8%)	
Natives	2 (2.4%)	0 (0.0%)	2 (5.1%)	
More than one races	1 (1.2%)	0 (0.0%)	1 (2.6%)	
Marriage	-	-	-	0.923
Single or Never Married	14 (17.1%)	8 (18.6%)	6 (15.4%)	
Separated, Divorced, Widowed	21 (25.6%)	11 (25.6%)	10 (25.6%)	
Married or Living with Partner	47 (57.3%)	24 (55.8%)	23 (59.0%)	
Education	-	-	-	0.534
Lower than High School	1 (1.2%)	0 (0.0%)	1 (2.6%)	
High School Graduate	3 (3.7%)	2 (4.7%)	1 (2.6%)	
Associate or Some College	24 (29.3%)	10 (23.3%)	14 (35.9%)	
College Degree	18 (22.0%)	11 (25.6%)	7 (17.9%)	
Graduate Degree or Above	36 (43.9%)	20 (46.5%)	16 (41.0%)	
Employment	-	-	-	0.471
Full-Time	58 (70.7%)	29 (67.4%)	29 (74.4%)	
Part-Time	3 (3.7%)	3 (7.0%)	0 (0.0%)	
Retired	15 (18.3%)	7 (16.3%)	8 (20.5%)	
Self-Own Business	3 (3.7%)	2 (4.7%)	1 (2.6%)	
Not working	3 (3.7%)	2 (4.7%)	1 (2.6%)	
Religion	-	-	-	0.103
Christian or Catholic	47 (57.3%)	21 (48.8%)	26 (66.7%)	
Others	35 (42.7%)	22 (51.2%)	13 (33.3%)	
No. Languages	1.50 (0.08)	1.44 (0.09)	1.56 (0.14)	0.469
Device Ownership (multiple choices)	-	-	-	
Smartphone	80 (97.6%)	42 (97.7%)	38 (97.4%)	0.944
Computer	73 (89.0%)	39 (90.7%)	34 (87.2%)	0.611
Tablet	56 (68.3%)	27 (62.8%)	29 (74.4%)	0.261
Smart Home Appliance	21 (25.6%)	12 (27.9%)	9 (23.1%)	0.617
No. of Devices (mean, std.)	2.80 (0.09)	2.79 (0.14)	2.82 (0.12)	0.869
Health Insurance Type (multiple choices)	-	-	-	
Medicare	22 (26.8%)	12 (27.9%)	10 (25.6%)	0.817
Medicaid or Other Government	4 (4.9%)	2 (4.7%)	2 (5.1%)	0.92
Private Insurance	64 (78.0%)	31 (72.1%)	33 (84.6%)	0.171
Dental Insurance	45 (54.9%)	23 (53.5%)	22 (56.4%)	0.791
Vision Insurance	39 (47.6%)	18 (41.9%)	21 (53.8%)	0.278
VA or Military	1 (1.2%)	1 (2.3%)	0 (0.0%)	0.338
Long-Term Care Insurance	7 (8.5%)	3 (7.0%)	4 (10.3%)	0.596
None	2 (2.4%)	2 (4.7%)	0 (0.0%)	0.173
No. Driving Days (min, max)	(0, 7)	(0, 7)	(3, 7)	
No. Driving Days (mean, std.)	5.95 (0.18)	5.91 (0.28)	6.00 (0.23)	0.798

The survey asked respondents about whom they took care of and two of them mentioned they were taking care of clients/patients. We removed these two cases given that they work for income and the type of care is determined by contract. Of the 80 respondents who care for their family members, including parents, grandparents, in-laws, spouses, or other relatives and friends, we further divided the sample into two groups: those taking care of people older than them, such as parents and grandparents (N=55, 68.8%), and those taking care of people at their age such as spouse (N=25, 31.2%).

Table 2 shows that emotional support is the top support for parents or grandparents (89.7% for the 0-4 group and 92.3% for the 5+ group), followed by medical support (N=46, 83.6%), support with daily activities (N=39, 70.9%), financial support (N=34, 61.8%), and lastly functional support (N=22, 40.0%). Additionally, the type of care provided does not vary significantly with the duration of caregiving (p>0.05), implying that the need for care remains constant over multiple years. Once individuals assume this responsibility, they tend to continue providing care in the long term.

**Table 2:** Types of Care by Parents or Grandparents by Years of Care

Parents or Grandparents	Total (N=55)	0-4 years (n=29)	5+ years (n=26)	p-value
Medical Support	46 (83.6%)	23 (79.3%)	23 (88.5%)	0.360
Functional Support	22 (40.0%)	12 (41.4%)	10 (38.5%)	0.825
Daily Activities	39 (70.9%)	20 (69.0%)	19 (73.1%)	0.737
Emotional Support	50 (90.9%)	26 (89.7%)	24 (92.3%)	0.733
Financial Support	34 (61.8%)	15 (51.7%)	19 (73.1%)	0.104

In Table 3, it is revealed that, both medical support and emotional support account for the majority of caregivers who take care of their spouses, relatives, or friends (N=18, 72.0%), followed by support with daily activities (N=16, 64.0%), financial support (N=13, 52.0%), and lastly functional support (N=10, 40.0%). The trends seen in both Table 2 and Table 3 are the same indicating that despite the age of care recipients and their relationships with caregivers, the nature and the type of support remain consistent throughout the years. Additionally, the type of care did not vary by years of care (p>0.05), indicating that the need for care is the same over multiple years. Once a person starts this responsibility, they will carry on for good.

**Table 3:** Types of Care by Spouse or Others by Years of Care

Spouse or Others	Total (N=25)	0-4 years (n=13)	5+ years (n=12)	p-value
Medical Support	18 (72.0%)	8 (61.5%)	10 (83.3%)	0.225
Functional Support	10 (40.0%)	6 (46.2%)	4 (33.3%)	0.513
Daily Activities	16 (64.0%)	8 (61.5%)	8 (66.7%)	0.790
Emotional Support	18 (72.0%)	8 (61.5%)	10 (83.3%)	0.225
Financial Support	13 (52.0%)	6 (46.2%)	7 (58.3%)	0.543

Content analysis results

The survey asked caregivers to describe the health issues that their care recipients have. Of the 80 respondents who provide unpaid care for their family members, we further divided the sample into two groups: those who have been caregivers for 0-4 years (N=42, 52.5%) and those who have been caregivers for 5+ years (N=38, 47.5%). However, given that some respondents reported multiple health conditions for their care recipients, the total number of health issues totaled up to 187, with 77 (41.2%) of them occurring within 0-4 years and 110 (58.8%) within the 5+ years caregiving group.

Table 4 shows that neurologic diseases, such as Parkinson’s disease, stroke, and Alzheimer’s disease, are the leading health conditions among care recipients, with 53 responses accounting for 28.3%. There is also a statistically significant difference between the 0-4 and 5+ years group with neurologic diseases (p<0.05). Functional support, such as for those with limited mobility and/or being wheelchair-bound, was the second most common response, with 31 responses constituting 16.6%. Cardiovascular diseases, with conditions such as hypertension, congestive heart failure, and coronary artery disease, are the third most common responses at 25, comprising 13.4%. The other reported health conditions include endocrine (diabetes), cancer, respiratory, musculoskeletal, genitourinary, metabolic, psychiatric, digestive, etc., accounting for 78 responses (41.7%).

**Table 4:** Health Conditions of Care Recipients by Years of Care

Health Conditions	Total (N=187)	0-4 years (n=77)	5+ years (n=110)	p-value
Functional Support	31 (16.6%)	14 (18.2%)	17 (15.5%)	0.676
Emotional Support	5 (2.7%)	2 (2.6%)	3 (2.7%)	0.731
Neurologic	53 (28.3%)	30 (39.0%)	23 (20.9%)	0.012
Cardiovascular	25 (13.4%)	7 (9.1%)	18 (16.4%)	0.167
Cancer	16 (8.6%)	10 (13.0%)	6 (5.5%)	0.203
Respiratory	10 (5.3%)	5 (6.5%)	5 (4.5%)	0.697
Musculoskeletal	11 (5.9%)	3 (3.9%)	8 (7.3%)	0.330
Endocrine	18 (9.6%)	5 (6.5%)	13 (11.8%)	0.294
Genitourinary	6 (3.2%)	1 (1.3%)	5 (4.5%)	0.329
Metabolic	5 (2.7%)	0 (0.0%)	5 (4.5%)	0.112
Others	7 (3.7%)	0 (0.0%)	7 (6.4%)	0.077

Resources needed

When we asked caregivers about what resources they needed to alleviate their financial burdens, resources to support their own physical and mental health, resources to take care of their loved ones, and resources to improve their quality of life, two prominent themes emerged from the responses: (1) medical related (63.8%) and (2) non-medical related resources (96.3%) (Table 5). Since some respondents reported more than one need but also some respondents did not think they needed any additional assistance, the total number of responses for this domain is 80. Table 5 also illustrates that a higher percentage of caregivers with more than five years of experience have medical-related needs than caregivers with fewer than five years of experience (69.2%>58.5%, p=0.244). On the contrary, caregivers with fewer than five years of experience have more non-medical related needs.

**Table 5:** Resources Needed for Caregiving by Years of Care

Types of Resource	Total (N=80)	0-4 years (n=41)	5+ years (n=39)	p-value
Medical Related	51 (63.8%)	24 (58.5%)	27 (69.2%)	0.244
Non-Medical Related	77 (96.3%)	41 (100.0%)	36 (92.3%)	0.276

Medical-related needs

Of 80 informal caregivers, 51 of them (63.8%) expressed the need for medical-related resources. Over half of these respondents (N=33, 64.7%) mentioned the challenges of high medication costs and difficulty accessing insurance coverage for necessary items.

“Eliquis is over 500 per month alone. Medicare will not pay. Had a hard time getting the physician to write a script for a hospital bed and had to pay over 200 per month for a period of time.” (Participant 42)

In addition, 25 caregivers expressed a strong desire to enhance their knowledge and understanding of their loved one’s health conditions. They recognized the importance of gaining deeper insights into these conditions to provide more effective and informed care.

“information about what to expect as dementia progresses, how to assist her, how to understand what is beyond her control and what she can do for herself” (Participant 10) 

“Learning more about Alzheimer’s and the rate of deterioration of the brain and how family can better care for them” (Participant 29)

**Figure 2:**
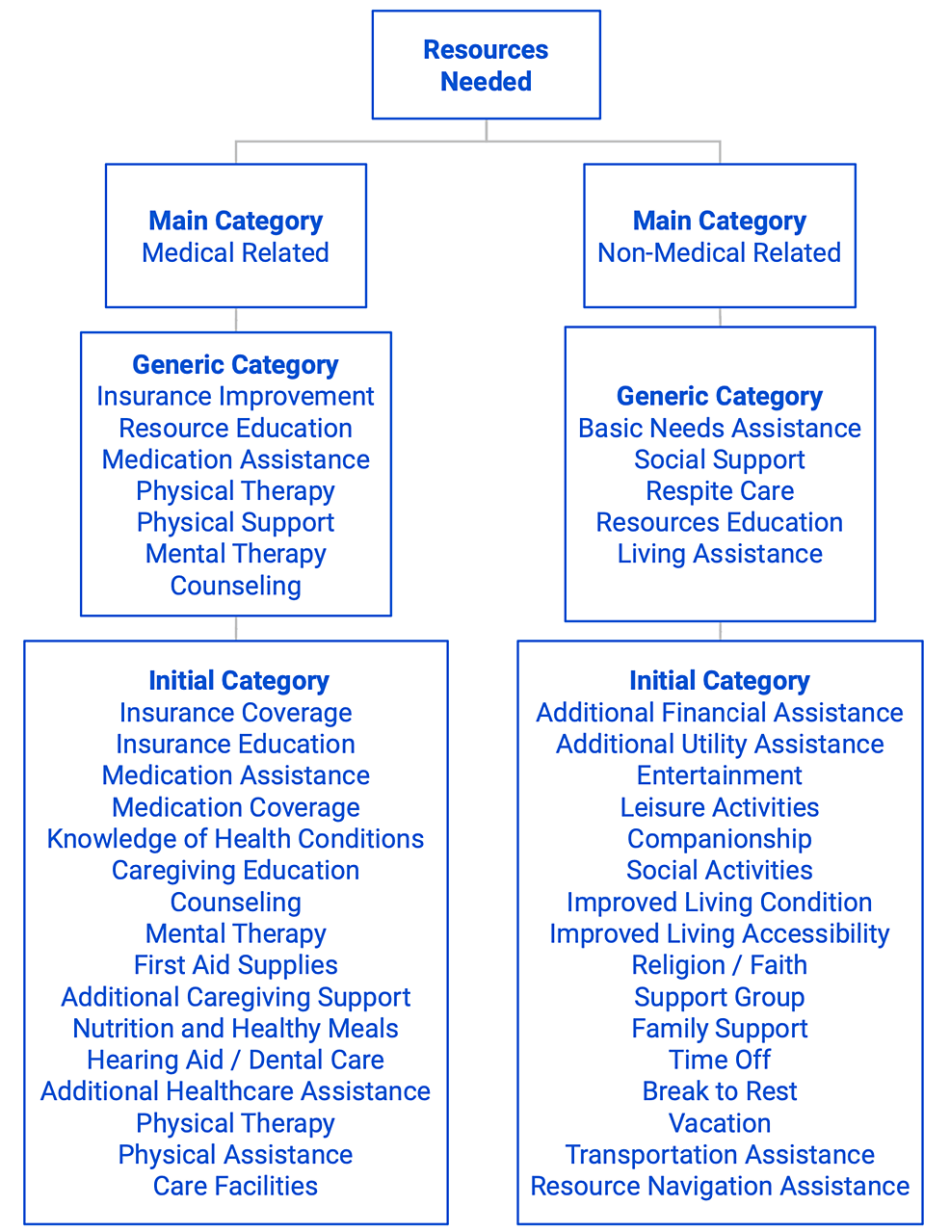
Resources Needed for Caregiving by Categories

Many participants also desired additional physical and mental therapy for caregivers and their care recipients to maintain their well-being. Caregivers recognized the need to maintain their health, acknowledging the benefits of regular exercise and therapy in coping with the demands of caregiving. The remaining participants expressed the need for general medical resources, including basic first aid supplies, additional healthcare professional assistance, knowledge about nutrition and how to cook healthy meals, etc. (Figure 2).

Non-medical related needs

On the other hand, the majority of caregivers expressed the need for non-medical related resources (N=78, 97.5%). Approximately 12 caregivers highlighted the need for essentials such as food, housing, utilities, transportation, and other necessities, indicating that caregivers face financial burdens beyond healthcare expenses.

“Assistance with Electric, Water, and Car Insurance to make paying Mortgage and have funds to pay for MEDS and FOOD.” (Participant 29)

Caregiving respite, which includes breaks for rest, vacations, and engagement in social and leisure activities, was a common response among caregivers. A substantial 45 or 56.3% of participants highlighted the necessity of respite to rejuvenate and recharge. Taking time off from caregiving responsibilities allows caregivers to prioritize their well-being, indulge in alone time and self-care, and engage in activities that bring joy and relaxation.

“time off from caregiving, ability to do things I like to do, keep my own health up, therapy” (Participant 33

“me time to just rest and relax without having to worry doctor’s appointment, ordering medication, making sure they have food to eat.” (Participant 38)

Caregiver support and education was another prominent theme, with 16 respondents (20%) emphasizing the need for additional caregiving help or daily home care living assistance to better support their loved ones and education on navigating different available resources to provide optimal care.

“what the social workers know - financial, medical, caretaking help resources” (Participant 9)

“learned that I need to be a full-time lawyer to navigate the volumes of information” (Participant 9)

The remaining caregivers expressed their needs for various non-medical resources, which include transportation, additional financial assistance, social and leisure activities, religion/prayer, support groups, and more. This emphasizes the wide-ranging and diverse needs of caregivers, reflecting the complex nature of their caregiving responsibilities. 

Figure 2 demonstrates a comprehensive list and detailed overview of the wide range of resources reported by caregivers. These resources were systemically categorized into smaller groups based on common themes and patterns that emerged from the survey responses. The "initial categories" reflect the prevalent themes derived from the majority of reported survey responses, which include insurance coverage, medication coverage, first aid supplies, social activities, vacation, religion, etc. These responses are further grouped into broader categories known as "Generic Categories" based on the common themes identified. These categories can include insurance improvement, resource education, social support, etc. Finally, the "Generic Categories" are divided into two main categories, medical-related and non-medical-related. 

Outcomes of care

Furthermore, to explore caregiving outcomes, we asked caregivers about their experiences regarding what they learned about themselves, their loved ones, different types of service, and the government’s role and cultural norms. Upon analysis of their responses, four prominent themes were identified: (1) changes in knowledge, care, and skills (77.5%), (2) changes in relationships (56.3%), (3) government policy and norms challenges (33.8%), and (4) knowledge about programs and services (40.0%) (Table 6). A higher percentage of caregivers with fewer than five years of experience indicate that they gained more knowledge in care (85.4%>69.2%) and related programs (48.8%>30.8%) than those with more than five years of experience. However, the variation is not statistically significant.

**Table 6:** Outcomes of Caregiving by Years of Care

Types of Outcomes	Total (N=80)	0-4 years (n=41)	5+ years (n=39)	p-value
Change in Knowledge, Care, and Skills	62 (77.5%)	35 (85.4%)	27 (69.2%)	0.394
Change in Relationship	45 (56.3%)	22 (53.7%)	23 (59.0%)	0.732
Government Policy and Norms Challenges	27 (33.8%)	12 (29.3%)	15 (38.5%)	0.377
Knowledge about Programs and Services	32 (40.0%)	20 (48.8%)	12 (30.8%)	0.158

Change in knowledge, care, and skills

Of 80 informal caregivers, 62 (77.5%) report change in knowledge, care, and skills and consequently gain valuable insights about themselves and their loved ones through their caregiving experience (Table 6). While caregiving is challenging, many experienced emotional and personal growth, from discovering their strengths, capabilities, weaknesses, and physical and emotional limitations to cultivating empathy and compassion. They also learned to recognize the importance of self-care and prioritize their own well-being, showcasing the transformative impact of the caregiving experience.

“That I am strong enough to endure more than I imagined; however, I have to also take care of myself because I didn’t realize how bad my burnout was getting.” (Participant 49)

On the other hand, some caregivers expressed feelings of being overwhelmed, frustrated, and exhausted by the unexpected demands, while some faced difficulties in fulfilling their caregiving responsibilities, including physical, emotional, and logistical challenges that they encounter on a daily basis.

“I get overwhelmed at times and I'm afraid of their end of life.” (Participant 68)

Additionally, many caregivers recognized the importance of early planning for their future, understanding the challenging aspects of caregiving, and learning about the impact of aging on both their physical and emotional well-being. This realization can be beneficial, prompting caregivers to prioritize proactive measures and seek knowledge to navigate the complexities associated with caregiving and aging.

Change in relationship

More than half of informal caregivers (N=45, 56.3%) report experiencing changes in their relationship with their loved ones in both positive and negative ways (Table 6). The caregiving role can evoke profound emotions, fostering a deep love, gratitude, and appreciation as caregivers learn more about their loved ones and their family history. Their responses revealed the optimism and resilience within the care recipients and emphasized the enduring bond of familial support.

“She has a way of finding the positive in any situation. She is always happy.” (Participant 10)

“I’ve learned that at times, he feels he is a burden to me, but I continue to ensure him that we are family and caring for one another is what family does.” (Participant 25)

On the other hand, caregiving can sometimes fracture relationships arising from disagreements, conflicts, and emotional tensions, especially when caregivers feel undervalued or unacknowledged for their expertise and efforts in providing care. Such situations can introduce tension and frustration within the caregiver-care-recipient relationship.

“That she can be stubborn and not give me credit for my knowledge and ability to take care of her.” (Participant 54)

Government policy and norms challenges

Table 6 demonstrates that 33.8% of caregivers also highlight the struggles and challenges they faced when navigating government resources, policies, and social norms and their lack of awareness of the systems. They expressed frustration with bureaucratic processes, complex regulations, and cultural and non-cultural barriers that hindered their ability to access the needed support.

“The lengthy process of being awarded disability even with a terminal illness” (Participant 49)

**Figure 3:**
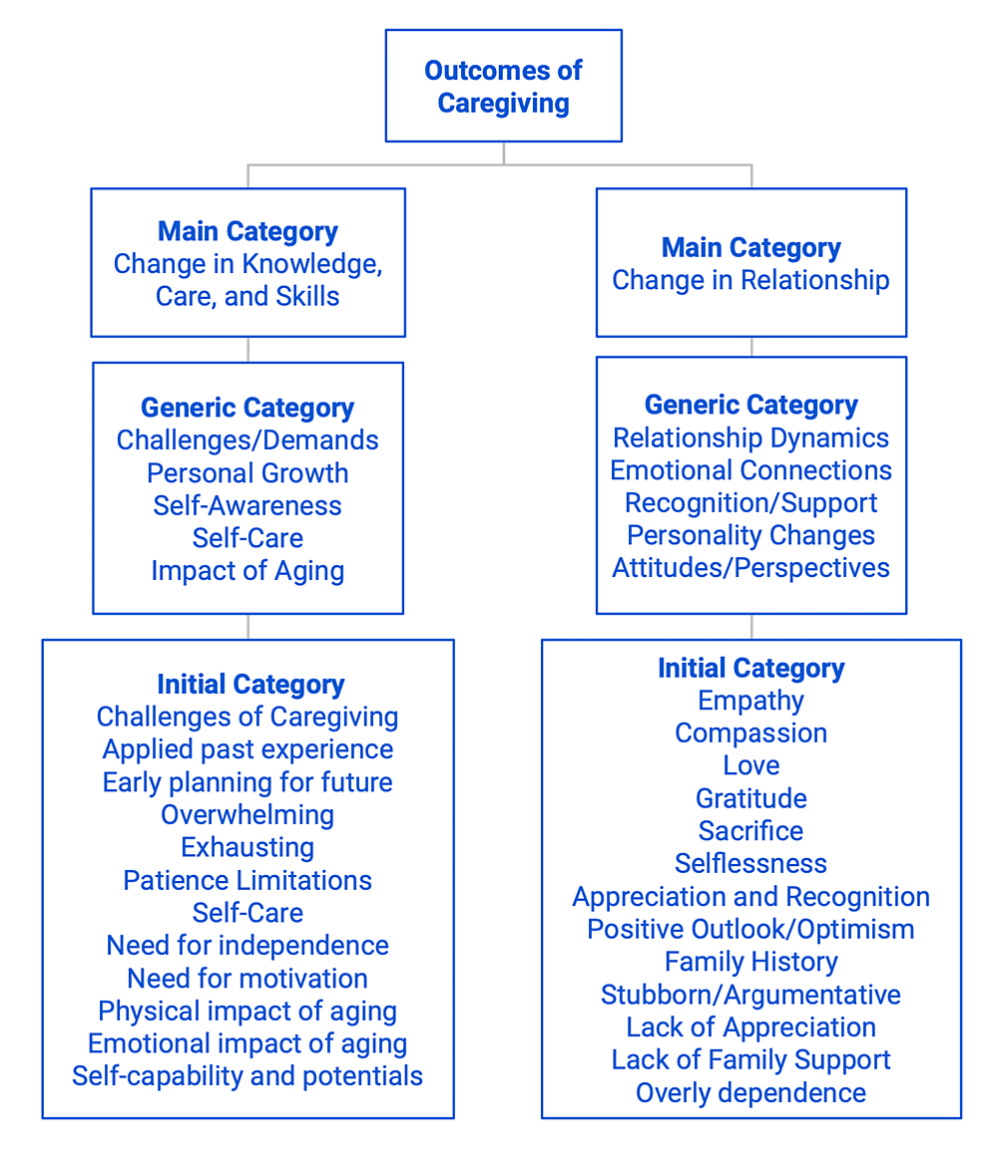
Outcomes of Caregiving by Categories, Part 1

**Figure 4:**
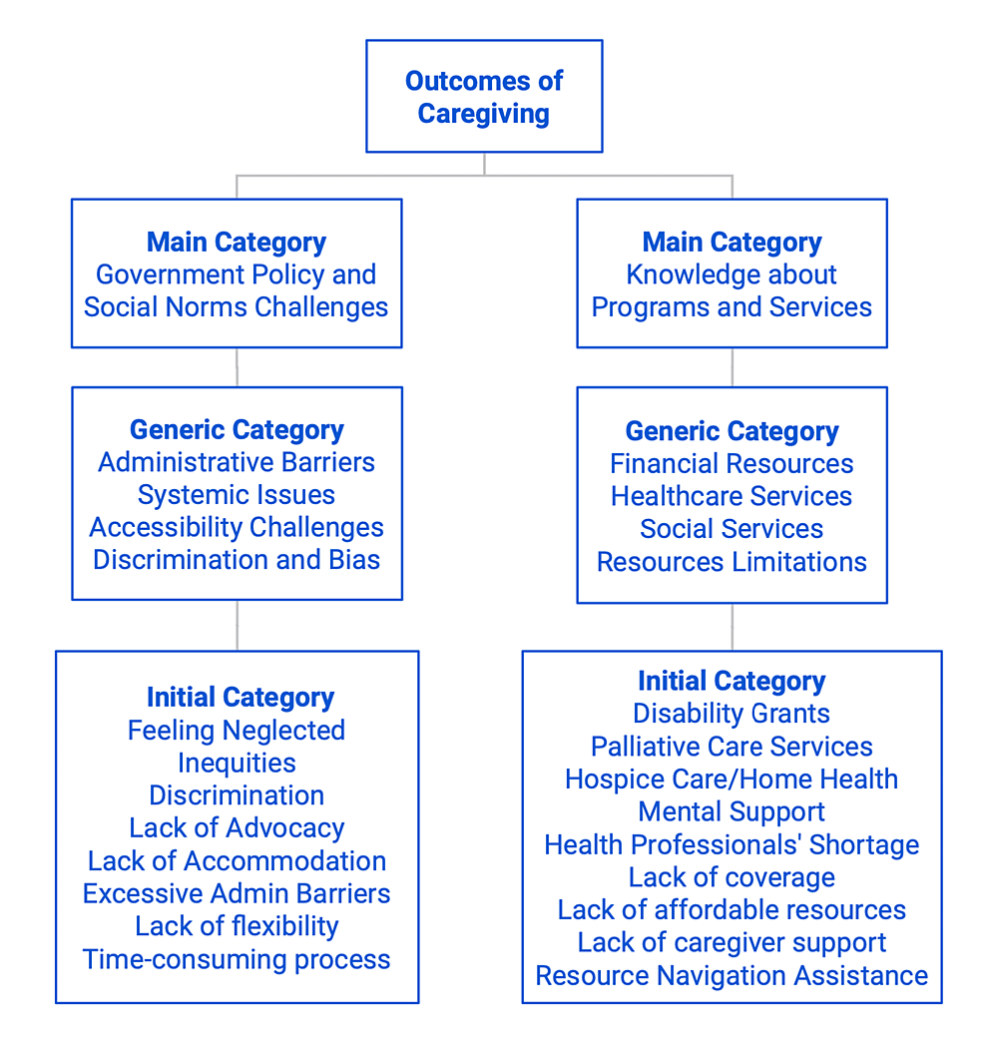
Outcomes of Caregiving by Categories, Part 2

They also experienced inequities, neglect, and inadequate support from the government, indicating a sense of dissatisfaction with the lack of support programs and affordable resources, limited coverage and assistance, and lack of accommodation and advocacy. A detailed list of phrases can be found in Figures 3-4.

“Poverty isn't as concerning to the Government as you would think, in respect to senior citizens.” (Participant 15)

Knowledge about programs and services

Lastly, 40.0% of caregivers reported that their caregiving experience led them to discover various services and programs available to assist them, ranging from financial resources to healthcare services and mental support. Not only did they become aware of these resources, but they also gained insights into their benefits and limitations.

“There are nowhere near enough things available to help senior citizens and no one has a clue how to help them” (Participant 78) 

“... I really wish there were more options for caregivers to both see that their family members are being taken care of in their own home and also allow the caregiver to have a full-time job (if they need/want) without financial stress and anxiety.” (Participant 45)

Figures 3 and 4 illustrate a comprehensive list and detailed overview of the wide range of outcomes of care reported by caregivers. These responses were systemically categorized into smaller groups based on common themes and patterns that emerged from the survey. The "initial categories" reflect the prevalent themes derived from the majority of reported survey responses, which include challenges of caregiving, family history, physical/emotional impact of aging, lack of advocacy, empathy, and compassion, etc. These responses are further grouped into broader categories known as "Generic Categories" based on the common themes identified. These categories can include personal growth, self-awareness, self-care, relationship dynamics, recognition and support, personality changes, etc. Finally, the "Generic Categories" are divided into four main categories, including change in knowledge, care, and skills; change in relationship; government policy and social norm challenges; and knowledge about programs and services. 

Thematic keyword analysis results

A comprehensive keyword analysis was conducted to identify significant themes from the survey responses. Table 7 illustrates the most prevalent keywords identified in the survey which include but are not limited to, “time, help, care, need, insurance, support, health, money, patience, home, health, Medicare, people, food.” These keywords reflect the core concerns expressed by the participants and provide valuable insights into their priorities and challenges.

**Table 7:** Survey Keyword Analysis by Frequency

Keywords	Frequency
time	34
care	31
patience	19
support	18
health	16
knowledge	14
Medicare	12
family	12
exercise	11
mental	10
help	9
elderly	9
financial	9
support group	9
dementia	8
medication	8
caregiver	8
socialization	7
assistance	7
resources	7
aging	6
home	6
transportation	6
support system	6
compassion	6
education	6

## Discussion

Through our analysis, we discovered that over 90% of informal caregivers are female, which closely aligns with the findings of a previous study indicating that middle-aged women constitute the majority of informal caregivers [19]. This information is particularly significant considering previous research showing that caregiving can be physically and emotionally burdensome to women working full-time [20] and that lacking work-family support has been found to be more strongly associated with exhaustion in women than in men [21]. These findings emphasize the pressing need for increased support and assistance for caregivers, particularly focusing on the unique challenges faced by women in balancing their caregiving duties with other responsibilities. Additionally, the age of caregivers ranged from 23 to 80 years old with a mean of 56 years old, indicating that caregivers are predominantly in their midlife to late adulthood stages. This is a period where many individuals may face their own health challenges while also simultaneously taking on caregiving responsibilities, making it particularly exhausting for them to handle. The wide range of ages can allow us to recognize the different unique needs they may have. For instance, younger caregivers may require assistance in balancing their caregiving duties with personal and professional commitments, while older caregivers might need more support on their well-being to prevent burnout.

Our study findings also show that the most prevalent health conditions in care recipients are neurologic diseases and functional impairment, which can pose significant physical and financial challenges for caregivers and eventually leads to psychological stress and poor quality of life. These complex and progressive conditions often require long-term care and specialized support to manage cognitive decline, motor impairments, and daily living challenges. Additionally, older adults experiencing limited to no mobility face many obstacles in carrying out routine activities independently, requiring ADL assistance to ensure their safety and optimize their quality of life. Cardiovascular disease also often requires medication management, lifestyle modifications, and monitoring of symptoms to ensure treatment compliance and prevent any life-threatening emergencies [22]. These prevalent health conditions present physical, mental, and substantial financial challenges for caregivers, who may also be caring for young children and other family members-the so-called “sandwich phenomenon” [23]. The costs associated with medical treatments, assistive devices, home modifications, and caregiving services can strain the financial resources of both caregivers and care recipients. The demanding nature of caregiving for individuals with these health conditions can lead to elevated stress levels, emotional strain, and a diminished quality of life for both caregivers and care recipients [24]. However, it is worth noting that the majority of health conditions did not vary significantly by years of care except for neurological diseases, suggesting that the length of caregiving provided may not directly influence the prevalence of specific health conditions among care recipients. Therefore, it is essential to consider that certain conditions often exhibit progressive characteristics and can be influenced by other factors over time. 

Furthermore, there is a greater need for non-medical than medical resources. However, our findings did not reveal any significant variation between these two types of resources. This underscores the importance of a comprehensive approach to caregiving and recognizes that both resources are crucial in meeting the diverse needs of caregivers and care recipients. Interestingly, Table 5 shows that the 5+ years group expressed a greater need for medical resources but a relatively lower need for non-medical care compared to the 0-4 years group. This pattern aligns with the expected trend of increased healthcare expenditure for any society with a growing aging population [25]. However, it is noteworthy that the overall resource needs did not significantly vary between the duration of care, which suggests that regardless of the duration of caregiving, caregivers continue to face similar demands and challenges, emphasizing the persistent need for resources throughout the caregiving journey.

Upon analyzing the outcomes of caregiving based on the duration of care, no significant differences were identified between caregivers with 0-4 years and 5+ years of experience across the identified themes, highlighting the transformative nature of the experience, the complexities of relationships, the challenges with government policies, and the importance of accessible programs and services regardless of the duration of care. For instance, 53.7% of caregivers with 0-4 years of experience and 59.0% of caregivers with 5+ years of experience report changes in relationships with their loved ones with no significant difference between the two categories, indicating that caregiving can quickly influence the relationships between both parties and will likely last beyond five years. Additionally, our findings indicate that more than half of the total caregivers (N=46, 57.5%) reported negative outcomes either in their relationships or overall experience with caregiving, while a smaller proportion (N=29, 36.3%) had positive experiences. Figure 3 provides examples of both positive and negative experiences. Positive experiences included empathy, compassion, love, gratitude, recognition, and appreciation, whereas negative experiences included stubbornness, arguments, and lack of appreciation, recognition, and support. These results underscore the urgent need for solutions that can enhance caregiving experiences, promoting a positive environment that cultivates gratitude and ensures consistent and quality care.

Furthermore, our study revealed caregivers’ struggles with government resources, policies, and societal norms with many of them facing frustrations due to complex bureaucratic processes, regulations, and cultural barriers, hindering their access to support. They often encountered difficulties understanding and navigating available resources, while some lacked awareness and guidance about support systems which further intensified their challenges, leaving them feeling lost and struggling to make informed decisions. The responses also shed light on inequities, neglect, and inadequate support from the government and state, dissatisfaction with the lack of comprehensive and accessible support, and exposing critical gaps which emphasize the urgent need for policy changes and improvements to address these gaps. Bureaucratic processes should be streamlined, regulations simplified, and barriers removed to enhance caregiver access to support.

The Centers for Disease Control and Prevention recognizes caregiving as a public health concern and urges public health professionals and the government to address and promote changes to the current systems to improve the health and well-being of both caregivers and their care recipients [6]. The recommendations emphasize the need to educate the public about caregiving resources and healthcare providers about caregiver health risks [6]. It is also important to evaluate caregiver support programs, estimate service demand, increase awareness of evidence-based services, promote caregiver self-care, and ensure access to self-management programs for caregivers with disabilities or chronic diseases [6]. One example of a successful intervention from the government is seen in the South-East region of France, where they implemented a Social Support Services (3S), a formal service developed to provide social support, e.g., household chores, ironing, shopping, or travel support, to retired individuals who are at risk of dependency [26]. However, the coverage provided by the program is income-dependent with varying financial contributions from 10% to 73% of the 3S cost [26]. Nevertheless, the study revealed that the implementation of a social assistance program had a positive impact and improved the burden on caregivers, emphasizing the importance of implementing evidence-based programs to support informal caregivers in our country [26].

Caregivers also faced challenges with the lack of accommodation and appropriate facilities and infrastructure for individuals with disabilities, as well as the unique needs of caregivers and care recipients. They emphasized the importance of designing indoor and outdoor environments that consider accessibility and address the specific requirements of individuals with disabilities. Additionally, the majority of caregivers are still in the workforce, which raised the need for extended business hours to after-hours and weekends to better accommodate their caregiving responsibilities. Many caregivers struggle to balance their work obligations with their caregiving duties, often facing challenges in finding suitable time slots for appointments, accessing necessary services, or attending support programs [27].

On the other hand, “time” was the most prevalent keyword identified among all survey responses analyzed, underscoring the central need expressed by caregivers. The participants consistently stressed the importance of respite opportunities, including the ability to take time off from caregiving responsibilities for personal well-being, vacation, solitude, and reconnecting with family and loved ones. Additionally, the analysis brought to light the theme of “support and help,” which underscored the significance of accessing various forms of support. Caregivers expressed the need for emotional support and physical assistance in managing the demanding tasks associated with caregiving, as well as financial assistance to address the financial strains often incurred in providing care. Moreover, the thematic analysis revealed the emergence of several other notable keywords, such as “patience,” “insurance,” and “money and food.” The theme of “patience” signifies the demanding nature of caregiving and the essential role patience plays in navigating the caregiving role. The keywords “insurance” and “money and food” point towards the financial implications and strains associated with caregiving responsibilities, highlighting the need for adequate support and access to resources that ensure the provision of essential needs for both the caregiver and the care recipient.

New technologies are constantly being developed and introduced into medical practice [28]. Technology has been revolutionizing health care and has the potential to play a significant role in supporting caregivers and alleviating some of the challenges they face [29]. A user-friendly smartphone app designed specifically for caregivers can provide a range of features and resources to support their caregiving responsibilities. The study's findings from identifying the services and resources caregivers need will guide us in improving our app. For instance, many caregivers expressed struggles with their physical and mental well-being, emphasizing the need for mental therapy, counseling, meditation, and physical exercise assistance to improve their overall health. Many of them also noted significant time constraints due to the demands of their caregiving, work, and family responsibilities. Thus, a tailored app that is accessible anywhere and anytime would be beneficial for caregivers. Moreover, financial burdens were a major concern for many caregivers. Knowing this information can help us design the smartphone app that offers solutions such as (1) meditation, good for mental well-being, (2) tai-chi, good for physical health, (3) prayer, good for spiritual health, and (4) free of charge, reduce financial burden. Furthermore, caregivers expressed the need for social interactions, activities, and companionship. These responses can help us explore a sharing function in the app which will allow users to share their experiences with friends or on social media, fostering a sense of connection and promoting mutual well-being. Encouraging joint use of the smartphone app by caregivers and care recipients can strengthen their bond and enhance their overall experience.

Study limitations

One limitation was the lack of knowledge and information regarding the severity of the care recipients’ conditions or diagnoses that shape the complexity of their needs. This has limited our understanding of the specific challenges caregivers face. Additionally, detailed information on the duration and length of conditions or diagnoses was unavailable, further constraining our analysis. Currently, limited literature explores the barriers and solutions related to English literacy and digital literacy among diverse users in the context of utilizing technology for caregiving purposes. Further research is needed to gain a deeper understanding of these challenges to enhance the development of a user-friendly smartphone app tailored to their circumstances and needs.

## Conclusions

Informal caregivers face many challenges that require support and assistance. Our study provides valuable insights into the burdens they experienced and reveals different resources they need to alleviate those burdens. Solutions are greatly needed to improve and make caregiving a more positive and meaningful experience, fostering continuity of care and allowing the care recipients to age at home. By understanding these burdens and the specific needs of caregivers, we can identify services and resources that can be substituted with technology. Hence, human caregivers can take a break from those tasks. It is crucial that caregiver education, stress reduction activities, and social care program access links be made more accessible and affordable for caregivers, such as through the use of our smartphone app, which caregivers can use at any time and any place. It is also essential to acknowledge and address the limitations of our study as they can potentially influence the scope and generalizability of our findings and the future design of other apps aimed at improving the quality of life of caregivers and care recipients.

## Figures and Tables

**Figure 5:**
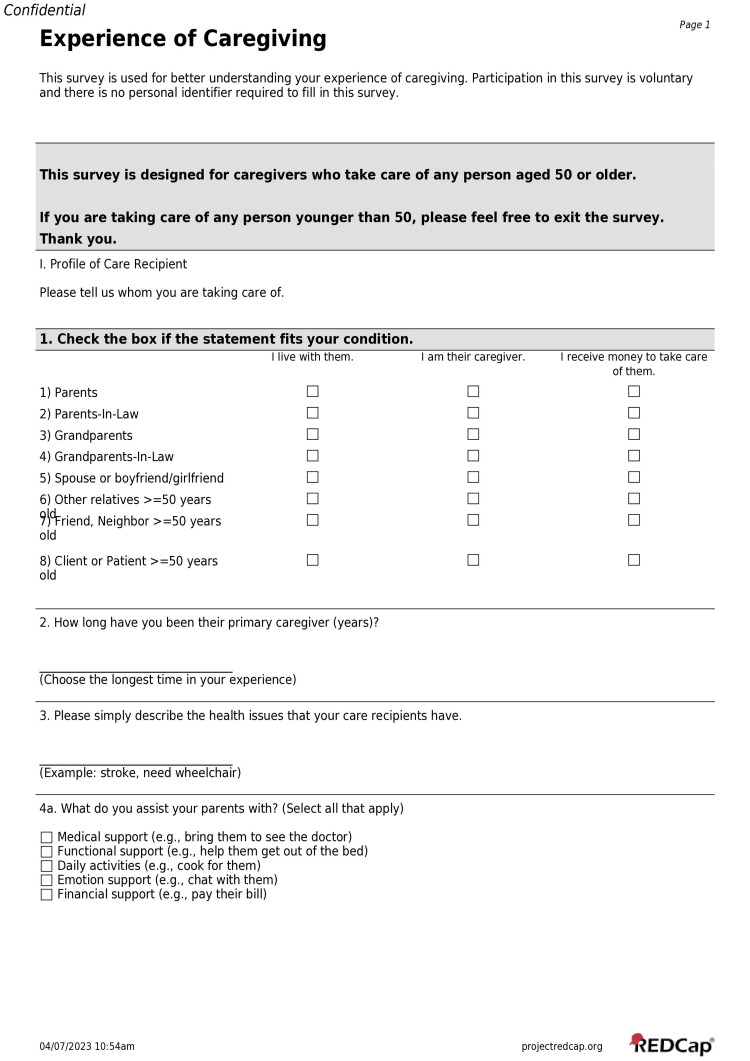
Experience of Caregiving Survey Questions, page 1

**Figure 6:**
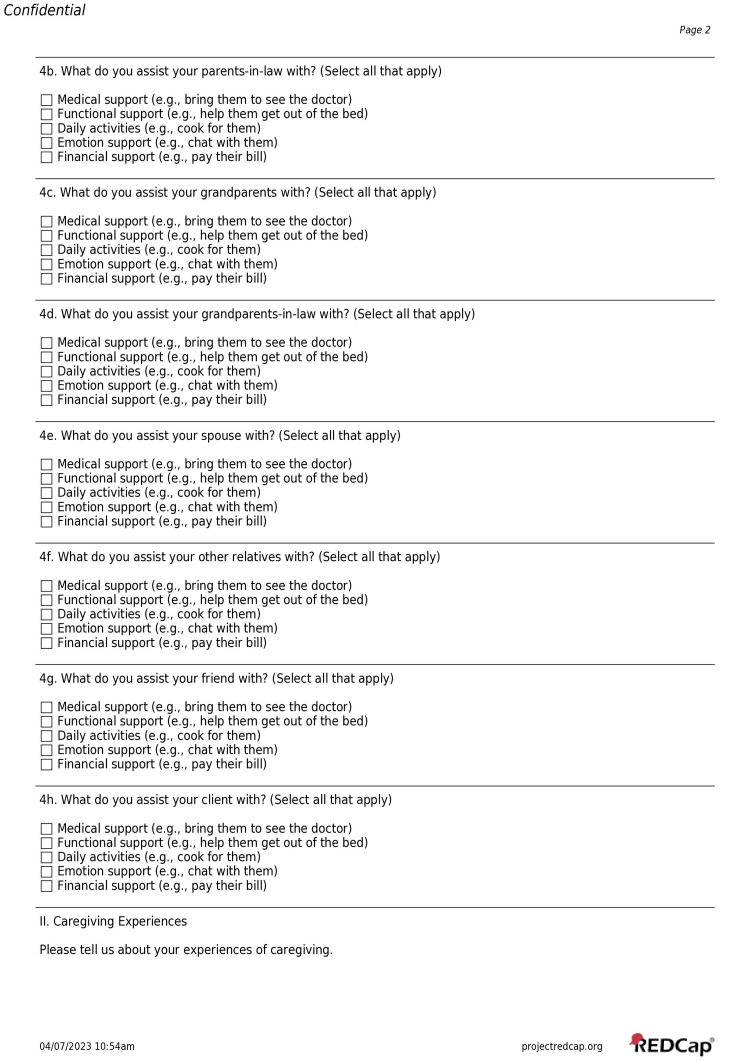
Experience of Caregiving Survey Questions, page 2

**Figure 7:**
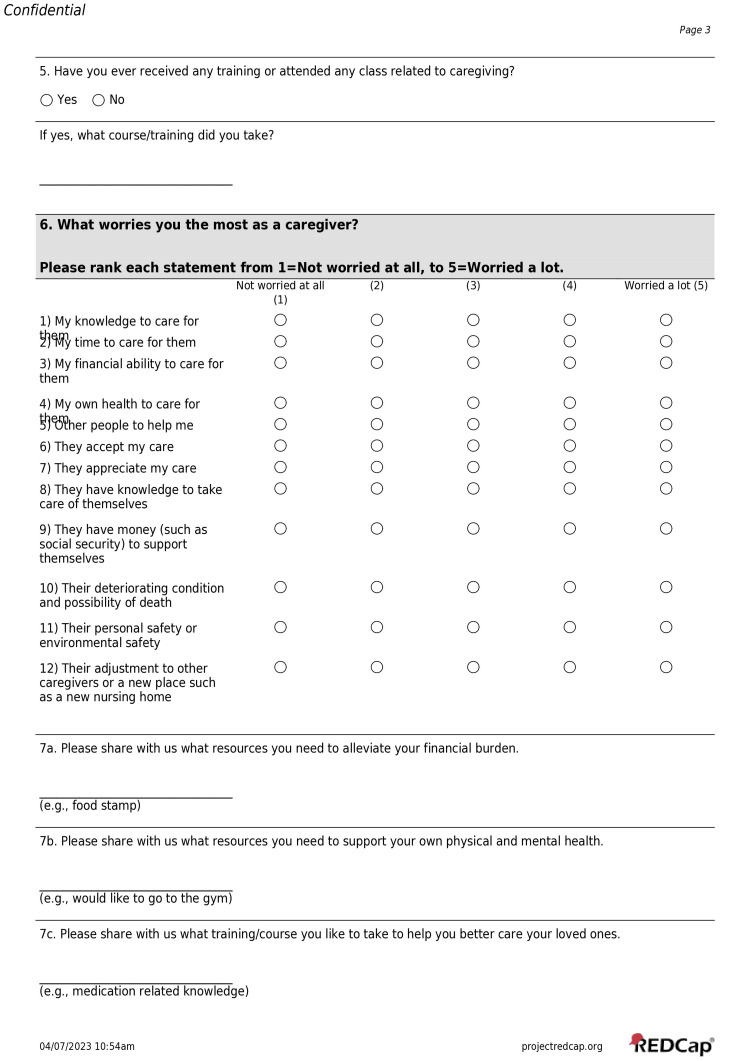
Experience of Caregiving Survey Questions, page 3

**Figure 8:**
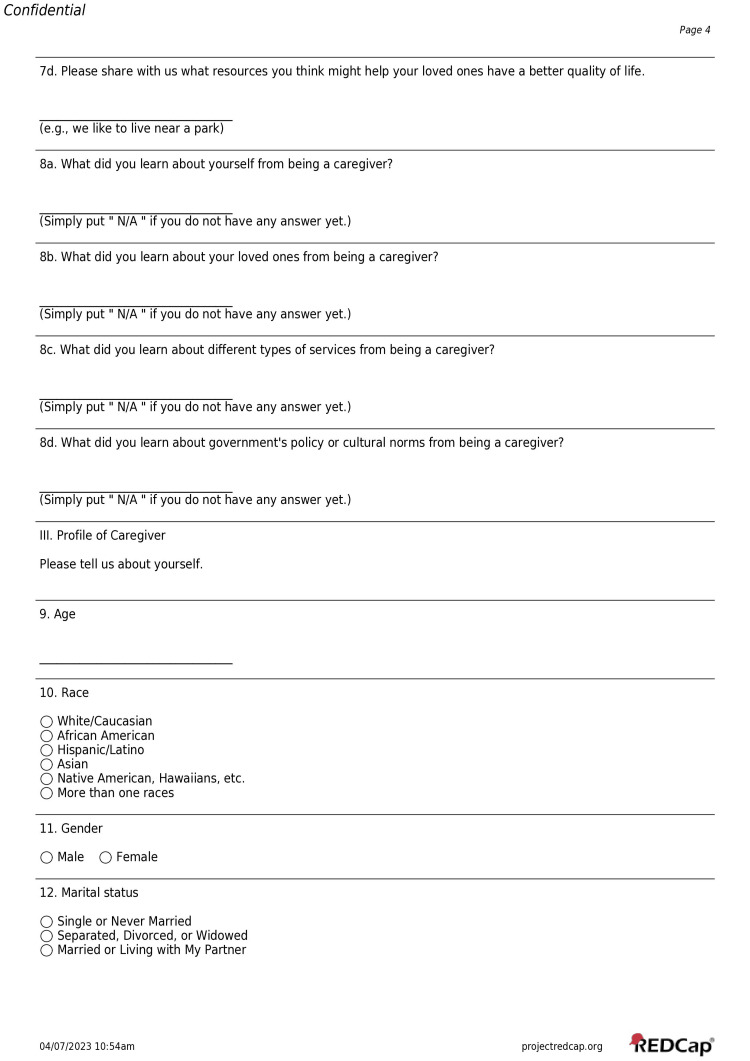
Experience of Caregiving Survey Questions, page 4

**Figure 9:**
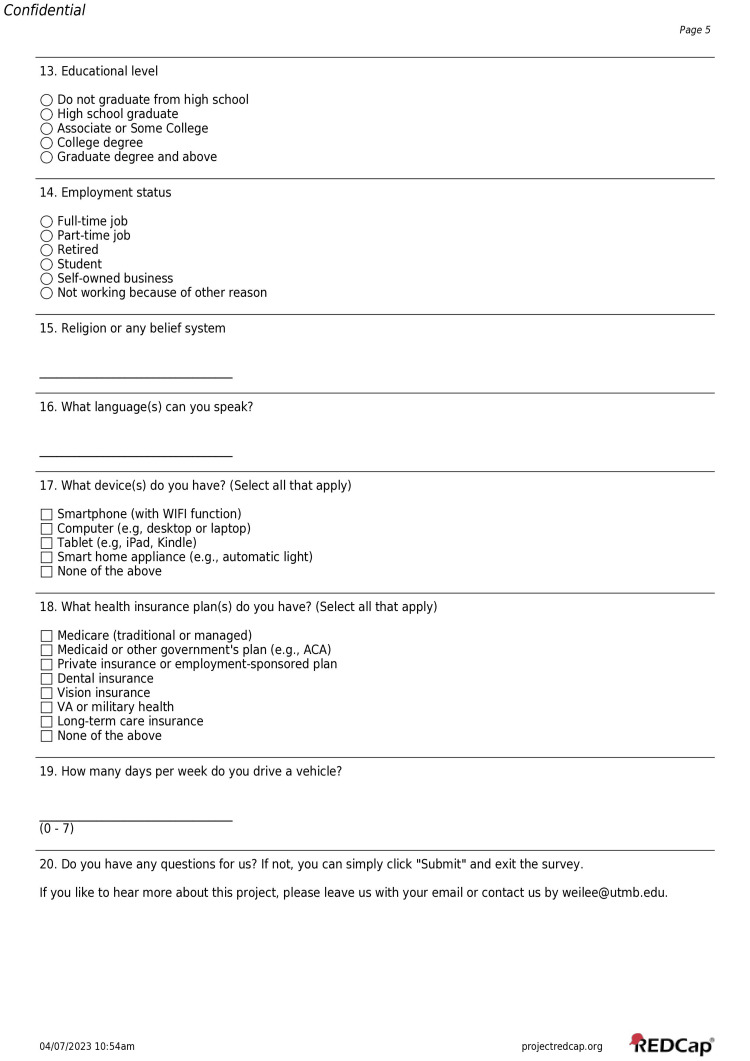
Experience of Caregiving Survey Questions, page 5

## Appendices

Experience of Caregiving Survey Questions
